# Test-retest properties of [^11^C]PXT012253 as a positron emission tomography (PET) radiotracer in healthy human brain: PET imaging of mGlu4

**DOI:** 10.1186/s13550-025-01266-y

**Published:** 2025-06-14

**Authors:** Per Stenkrona, Ryosuke Arakawa, Jiamei Guo, Benny Bang-Andersen, Sangram Nag, Mohammad Mahdi Moein, Zhisheng Jia, Zsolt Cselenyi, Christer Halldin, Andrea Varrone

**Affiliations:** 1 Department of Clinical Neuroscience, Karolinska Institutet and Centre for Psychiatry Research, Stockholm County Council, Stockholm, S-17176 Sweden; 2 H. Lundbeck A/S, Ottiliave j, Valby, 2500 Denmark; 3 Department of Psychiatry, The First Affiliated Hospital of Chongqing Medical University, Chongqing, 400016 P. R. China; 4 Department of Biophysics and Radiation Biology, Semmelweis University HUN-REN TKI, Budapest, 1094 Hungary

**Keywords:** Positron emission tomography (PET), Test-retest, Human brain, [^11^C]PXT012253, mGlu4

## Abstract

**Background:**

The metabotropic glutamate receptor 4 (mGlu4) has been proposed as a target for Parkinson’s disease to measure levodopa-induced dyskinesia. [^11^C]PXT012253 is a PET radioligand for mGlu4 (3.4 nM), previously characterized in non-human primates. We aimed to determine the optimal method for quantification, duration for acquisition, and test-retest reliability of the binding parameters for [^11^C]PXT012253 in healthy volunteers.

**Results:**

Six subjects (4 females) completed. [^11^C]PXT012253 displayed high uptake and rapid wash-out. Unchanged [^11^C]PXT012253 at 20 min was 10–20%. *V*_T_ in subcortical regions was higher than in cortical regions. 2TC provided better fits than 1TC. *V*_T_ by Logan GA and MA1 analysis correlated with that of 2TC-CM. MA1 showed better identifiability and standard error than Logan. The test-retest metrics in pons, putamen and thalamus showed absolute variability of *V*_T_<7% and ICC > 0.93 using the 2TC, Logan and MA1 graphical analyses. Time stability analysis showed that *V*_T_ values estimated using 63 min of imaging were within 10% of the values obtained with 93 min with all three models.

**Conclusion:**

[^11^C]PXT012253 showed a high brain uptake, with rapid washout and metabolism. *V*_T_ was reliably estimated using 2TC, Logan GA and MA1. The test-retest metrics showed high repeatability, indicating [^11^C]PXT012253 to be a suitable PET radioligand for mGlu4.

**Supplementary Information:**

The online version contains supplementary material available at 10.1186/s13550-025-01266-y.

## Introduction

L-Glutamate is the major excitatory neurotransmitter in the mammalian CNS. It acts via two classes of receptors, ligand gated ion channels (ionotropic receptors) and G-protein coupled (metabotropic) receptors (mGluR). mGluRs are expressed in virtually every major brain region at the presynaptic site of chemical synapses and act as fine tuners of the chemical transmission [1]. This occurs at excitatory (glutamatergic), inhibitory (GABAergic), and neuromodulatory synapses (monoamines, ACh, peptides) [2, 3]. mGluRs are subclassified into three groups based on sequence homology, G-protein coupling, and ligand selectivity. Group I include mGluRs 1 and 5, Group II includes mGluRs 2 and 3, and Group III includes mGluRs, 4, 6, 7, and 8. mGlu4 is expressed in the basal ganglia, which suggest a putative target for the treatment of neurological and psychiatric disorders such as Alzheimer’s disease, Parkinson’s disease, anxiety, depression, and schizophrenia [2, 4]. Modulation of presynaptic mGlu4 by an allosteric ligand has been proposed as a promising therapeutic target in Parkinson’s disease (PD) and levodopa-induced dyskinesia [5, 6].

The first clinical mGlu4-positive allosteric modulator (PAM) was recently tried in PD patients (PXT002331) [7]. Consequently, there is an increasing demand for the development of novel PET radioligands targeting mGlu4. With the successful development PET radioligands for mGlu1 and mGlu5, the utility of radioligands has been demonstrated in research studies with [^18^F]FIMX for mGlu1 [8]; [^11^C]ABP688, [^18^F]SP203 and [^18^F]FPEB for mGlu5 [9 - 11], and in clinical trials with [^18^F]FPEB [12, 13] and [^11^C]ABP688 for mGlu5 [14, 15].

Various chemotypes of mGluR4 positive allosteric modulators (PAMs) have been developed. The first PET imaging ligand for mGluR4 was a carbon-11 labeled derivative of N-(methylthiophenyl) picolinamide (^11^C-11 or ^11^C-KALB012) [16] later named [^11^C]PXT012253 [17, 18]. It has high affinity to an allosteric site of mGlu4 (3.4 nM) and has been characterized in rodents and in non-human primates (NHP) [6, 16, 18]. Our previous research in NHPs found the brain uptake of PET [^11^C]PXT012253 radiotracer was high, with a rapid washout [18]. Test-retest variability of the *V*_T_ was 17%. It was uniformly blocked by an analogue PXT002331, in all grey matter regions. These results indicated that [^11^C]PXT012253 might be a promising PET radioligand for measuring receptor occupancy of mGlu4 allosteric modulators in vivo.

To determine the suitability of [^11^C]PXT012253 application in clinical PET studies of central mGlu4, we carried out a positron emission tomography (PET) study in healthy volunteers. The aims were to determine the brain distribution of [^11^C]PXT012253, to identify the most reliable model to quantify [^11^C]PXT012253 radio tracer binding, and to estimate the intra-subject repeatability of [^11^C]PXT012253 binding in the brain. The validation of [^11^C]PXT012253 as a PET radioligands for the mGlu4 will enable to study mGlu4 binding in vivo in patients, e.g. levodopa-induced dyskinesia in Parkinson’s disease and as a target for novel drugs in Schizophrenia and depression.

## Materials and methods

### Ethical standards

The ethical standards in conducting the present study were in accordance with the Declaration of Helsinki and the European Medicines Agency “Guideline for good clinical practice E6(R2)”.

### Participants and study design

The healthy participants were recruited by advertisement on social media. The study protocol was approved by the Swedish ethical review authority and the Swedish Medical Product Agency. Written informed consent was obtained from all the participants. All the subjects met the inclusion/exclusion criteria (see Appendix 1). Each subject underwent a screening visit, a structural Magnetic Resonance Imaging (sMRI) scan, and two PET measurements. The two PET measurements were performed on the same day and within 30 days from the sMRI. Subject-specific plaster helmets were constructed to ensure minimal head movement during the PET measurements.

### PET measurements

The synthesis of [^11^C]PXT012253 was performed as previously reported [19]. All PET measurements were performed on the same PET-system, HRRT (Siemens/CTI), which has an effective resolution of 1.5 mm full width half maximum (FWHM) at the centre of the field of view (FOV). Before each PET measurement, a transmission scan of 6 min was performed with an external source of ^137^Caesium emitting gamma rays for attenuation correction of the PET images. The PET measurement was performed immediately after bolus injection of the [^11^C]PXT012253 followed by the i.v. injection of 20 mL physiological saline. Emission data was collected in list mode for 93 min. The list mode data was reconstructed into a series of 3D radioactivity images consisting of 38-time frames (9 × 10 s + 2 × 15 s + 3 × 20 s + 4 × 30 s + 4 × 60 s + 4 × 180 s + 12 × 360 s = 5580 s). The midpoint of each frame was used for the measurement of the mean concentration of radioactivity at each time point.

### Arterial blood sampling

Subjects had a catheter inserted in the radial artery for arterial blood sampling and a venous catheter inserted in the antecubital vein for radiotracer injection. An Automatic Blood Sampling System (ABSS) was used to withdraw arterial blood for 10 min, to measure radioactivity in the arterial blood every second after injection of [^11^C]PXT012253. In addition, manual samples of arterial blood were taken at 2, 4, 6, 8, 10, 15, 20, 30, 45, 60, 90 min to measure radioactivity in whole blood and for radiometabolite analysis. The manual samples were centrifuged to measure for radioactivity in plasma. Radiometabolite analysis was performed as previously reported [18, 19].

### Image analysis

PET images were analysed using an in-house developed image analysis pipeline. First, the sMRI image was reoriented so that the axial image was parallel to the plane having the anterior and posterior commissures. The reoriented sMRI was coregistered to the mean PET image saving the transformation parameters (coregistration matrix). Regions of interest (ROIs) were defined by an Anatomical Automatic Labeling (AAL) template on each individual sMRI scan. The MRI images were segmented into grey and white matter masks. The GM mask was used to mask the AAL templates to eliminate any overinclusion by the ROIs. The ROIs selected for the quantitative analysis were thalamus, putamen, frontal cortex, cerebellum and pons. The masked ROIs was projected and resliced to the space of the corresponding PET image using the coregistration matrix. The resliced ROIs were applied to the dynamic PET images to obtain the Time-activity curves (TACs) expressed as Standardized Uptake Values (SUV) calculated by ratio between tissue radioactivity concentration (kBq/cc) and administered radioactivity (MBq), divided by body weight (kg).

### Kinetic model analysis

The kinetic modelling was performed using the PMOD 3.4 software package (PMOD Group, Zurich, Switzerland). The outcome measure was the total distribution volume (*V*_T_) of [^11^C]PXT012253 for each ROI, applying different models and using the metabolite-corrected arterial input function. The models implemented were the one-tissue and two-tissue compartment models (1TCM, 2TCM), Logan graphical analysis (Logan) [20] and the multilinear analysis (MA1) [21]. Reliability among the models was estimated by comparing the Akaike information criterion (AIC) and the Model Selection Criterion (MSC). Reliability of the graphical analyses (Logan and MA1) was estimated by comparing percent Standard error of *V*_T_. The absolute intra-subject variability (VAR) of the *V*_T_ was calculated by the absolute difference divided by the average difference of the two examinations according to:

$$\:\text{V}\text{A}\text{R}=\:\frac{\left|\text{V}\text{T}\:\text{P}\text{E}\text{T}1-\text{V}\text{T}\:\text{P}\text{E}\text{T}2\right|}{\left(\text{V}\text{T}\:\text{P}\text{E}\text{T}1+\:\text{V}\text{T}\:\text{P}\text{E}\text{T}2\right)}*200$$

The intraclass correlation coefficients (ICC) of the *V*_T_s were calculates as a relative measure of the reliability in *V*_T_ where the test and retest variability of *V*_T_ within subjects (*MS*_*W*_) was related to the variability of that among the subjects (*MS*_*B*_) according to the following equation:

$$\:\text{ICC}=\frac{\text{MS}_\text{B}-\text{MS}_\text{W}}{\text{MS}_\text{B}+\left(k-1\right)\text{MS}_\text{W}}$$

### Statistical analysis

Statistics analysis was performed with the software JMP^®^, Version 18.2.0. SAS Institute Inc., Cary, NC, 1989–2023. Descriptive analysis was performed for the *V*_T_ values. Statistical testing with Oneway Anova was performed for the time stability of *V*_T_ and of absolute *V*_T_ variability values.

## Results

### Demographic and exposure data

In total, 6 subjects were included (4 females). Table 1 shows a summary of demographic details and amount of injected [^11^C]PXT012253. Mean (range) of injected radioactivity 373 MBq (297–539), molar radioactivity 1090 GBq/µmol (696–2214), injected mass 0.11 µg (0.05–0.18).

**Table 1:** Demographic details of subjects and injected radioactivity and mass

			PET 1		PET 2	
Gender (M/F)	Age (yr)	BMI (kg/m^2^)	Injected radioactivity (MBq)	Mass (µg)	Injected radioactivity (MBq)	Mass (µg)
M	21	25.7	510	0.16	539	0.18
M	21	20.5	398	0.08	386	0.05
F	21	22.9	297	0.11	334	0.13
F	24	21.9	337	0.08	350	0.01
F	22	21.5	335	0.10	364	0.13
N	30	20.2	310	0.09	316	0.07
Mean ± SD	23.2 ± 3.52	22.1 ± 2.0	365 ± 79	0.10 ± 0.03	382 ± 81	0.11 ± 0.05

### Regional brain distribution of [^11^C]PXT012253

**Fig. 1:**
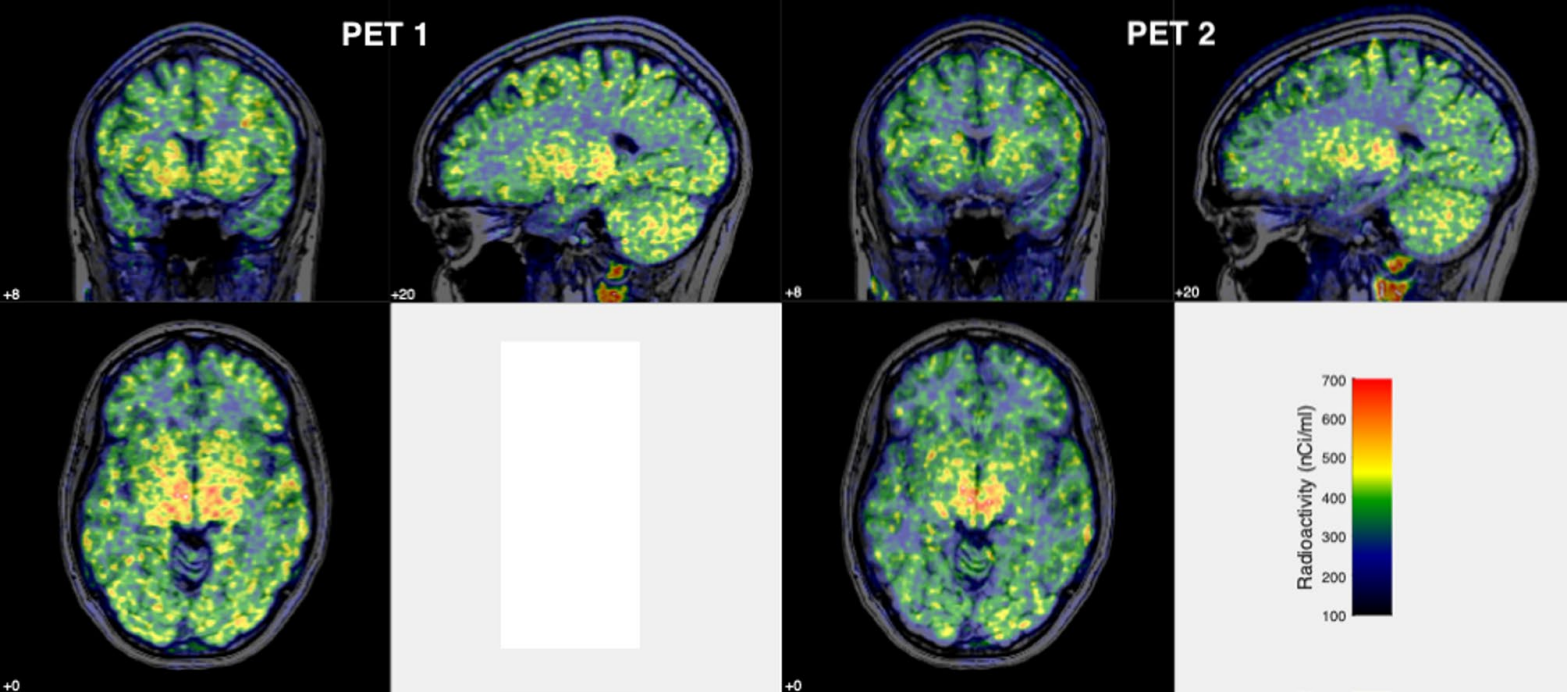
Summation PET images (AUC, 0–30 min), radiographic view, superimposed on corresponding MRI images in a healthy volunteer after i.v. injection of [^11^C]PXT012253, at two different occasions (**A** and **B**)

The AUC summation 0–30 min images demonstrated a widespread uptake of radioactivity in the brain (Fig. 1).

**Fig. 2:**
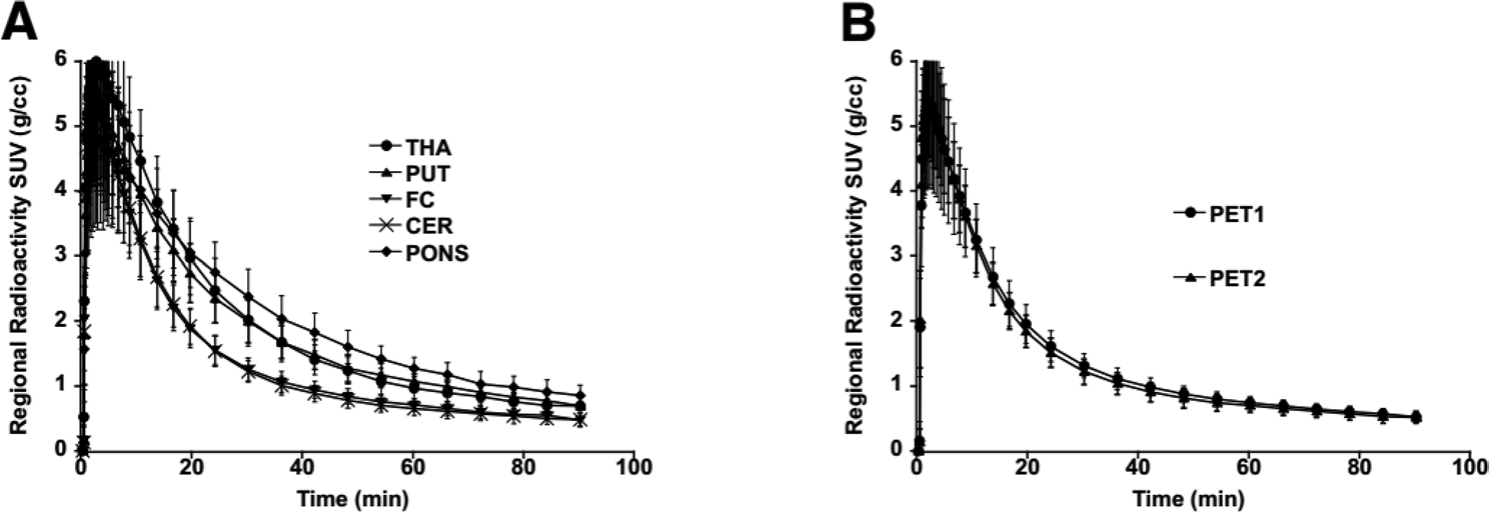
The mean time-activity curves for PET1 (*n* = 6; **A**), and the mean (SD) grey matter time-activity curves for PET1 and PET 2 (*n* = 6; **B**). THA: thalamus, PUT: putamen, FC: frontal cortex, CER: cerebellum, PONS

The regional TACs showed a rapid uptake and washout of [^11^C]PXT012253 in the grey matter regions (Fig. 2). The slowest washout was observed in the pons, followed by the thalamus, striatum, cortex and cerebellum (Fig. 2A). The washout of [^11^C]PXT012253 in the white matter (WM) was slow and after 45 min the TAC of [^11^C]PXT012253 in the WM was higher than those of all grey matter regions (see Supplements).

TACs in the average grey matter were similar for PET1 and PET 2 (Fig. 2B). The mean (± SD) peak SUV in the whole brain for the six subjects was 5.5 (± 0.8) for PET1 and 5.4 (± 0,5) for PET2, with the total range of 4.3–6.3.

**Fig. 3:**
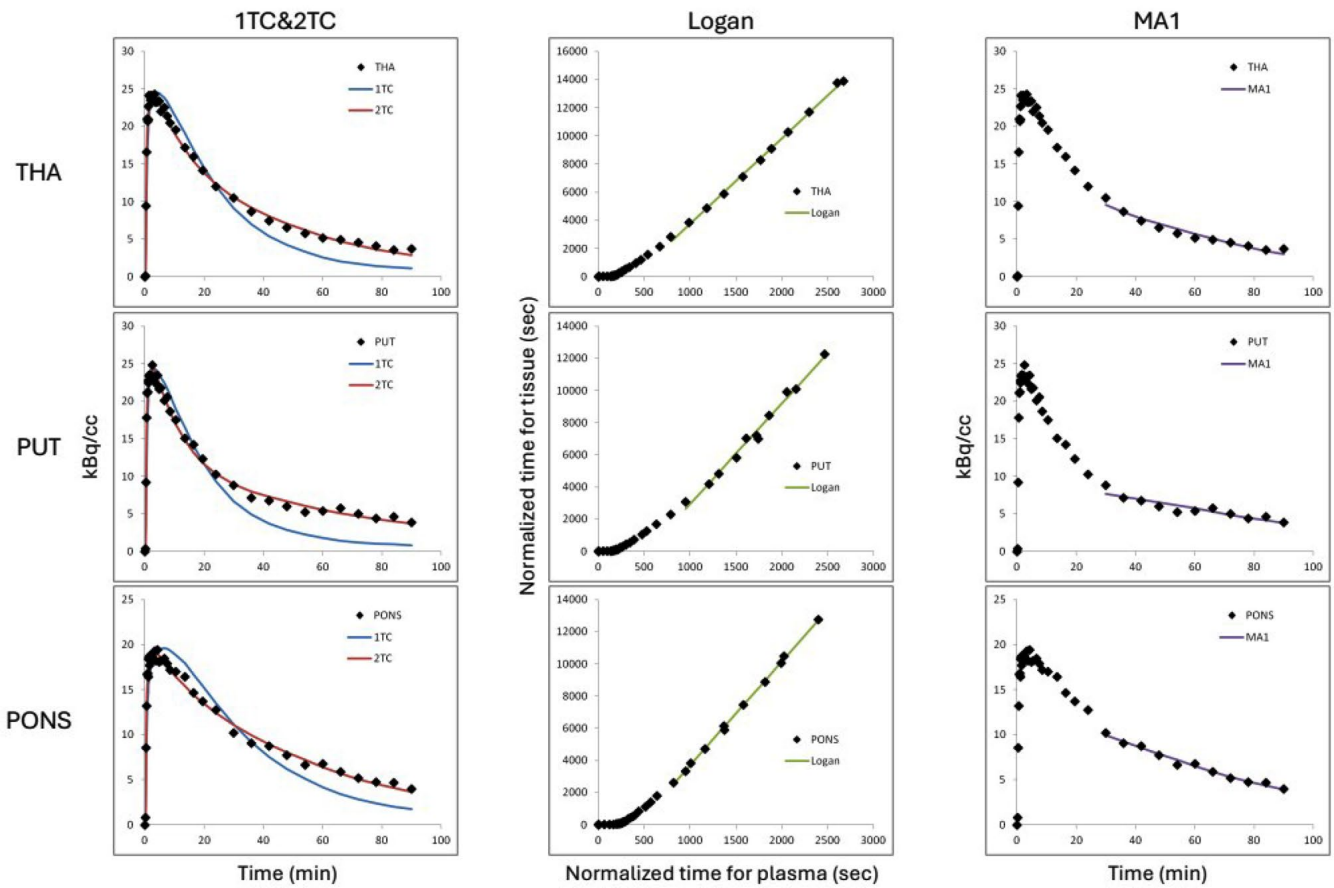
The curves of model fitting in the thalamus, putamen, and pons using 1TC & 2TC, Logan, and MA1 models (Subj. 1, PET 1)

The curves of model fitting in the thalamus, putamen and pons as representative regions are shown in Fig. 3. 2TC, Logan and MA1 analysis provided good on visual inspection, whereas 1TC showed poor fitting. Estimates of the *V*_T_ for all selected regions, based on the four different modelling approaches are shown in Table 2. *V*_T_ was high in pons, medium in thalamus and striatum, and low in cortex and cerebellum.

**Table 2:** Mean and SD of *V*_T_ in selected regions (mL/ccm)

	1TC	2TC	Logan	MA1
THA	4.4 ± 0.8	5.5 ± 0.9	5.7 ± 0.9	5.6 ± 0.9
PUT	3.6 ± 0.7	5.3 ± 1.1	5.4 ± 1.0	5.4 ± 1.0
FC	2.6 ± 0.5	4.0 ± 0.8	4.3 ± 0.9	4.2 ± 0.9
CER	2.7 ± 0.5	3.6 ± 0.6	4.0 ± 0.7	4.0 ± 0.7
PONS	5.0 ± 1.0	6.1 ± 1.0	6.2 ± 1.1	6.2 ± 1.1

The 2TC showed better fitting than the 1TC based on lower Akaike information criterion (AIC; 9,8 ± 7,7 vs. 81 ± 3,9, Mean ± SD) and higher model selection criterion (MSC; 4.7 ± 0.1 vs. 2.2 ± 0.3, Mean ± SD) (Fig. 3). As for the graphical models, MA1 showed better identifiability than Logan, as smaller standard error (1.2 vs. 1.7). t* of Logan and MA1 was set at 27.0 min for all subjects and regions. First, t* was estimated as 27.0 min when Max Err was 10% in the whole brain of most subjects. Next, *V*_T_ was calculated when t* was fixed at 27.0 min for all subjects and regions

**Fig. 4:**
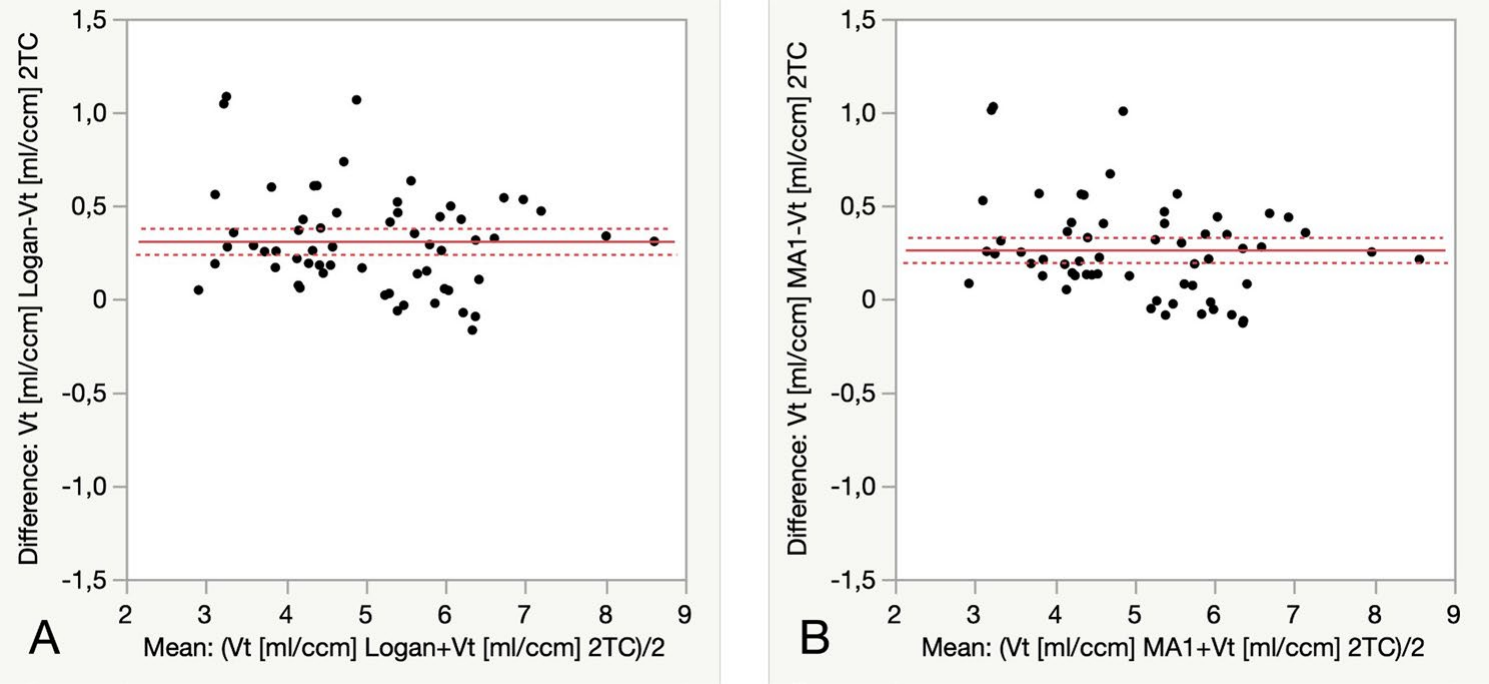
Bland-altman plots showing significant overestimation of *V*_T_ by graphical analysis, Logan 7.3 (± 8.0) % (**A**) and MA1 6.4 (± 7.8) % (**B**) (Mean ± SD; *p* < 0.0001 respectively) compared to 2TC in the five selected regions (Thalamus, Putamen, Frontal Cortex, Cerebellum and Pons) in the six subjects, each with two PET examinations

Logan and MA1 were well correlated with the 2TC for *V*_T_ values although a slight overestimation was observed in both models (Fig. 4).

### Radiometabolite analysis

**Fig. 5:**
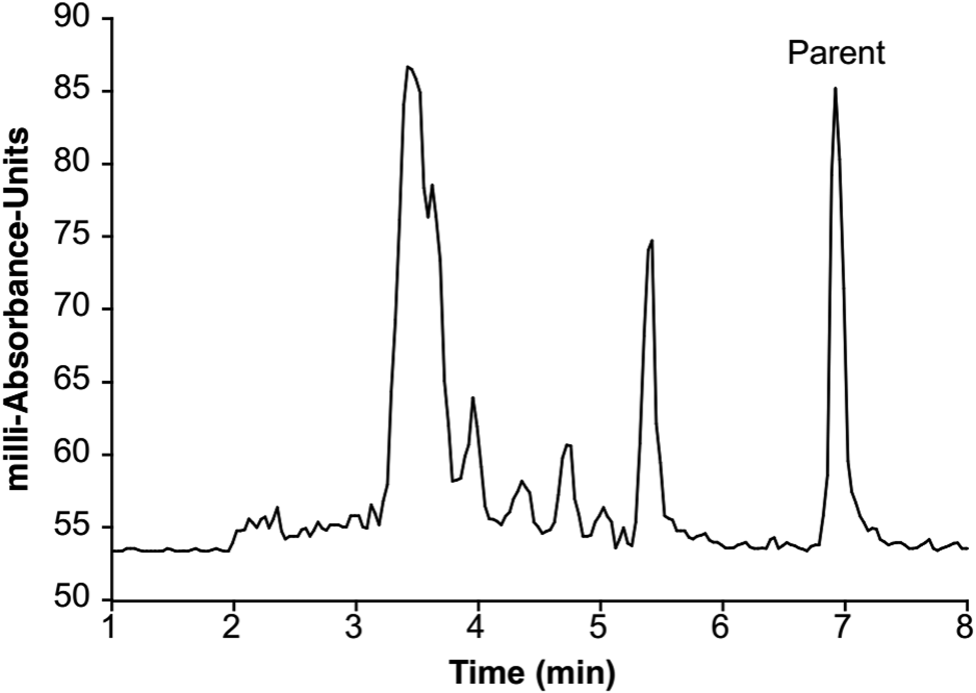
Radio-HPLC chromatogram (S101 PET1 20 min sample)

The HPLC chromatograms demonstrated that the major radiometabolite were less lipophilic than that of the parent compound making them less likely to enter the brain (Fig. 5).

**Fig. 6:**
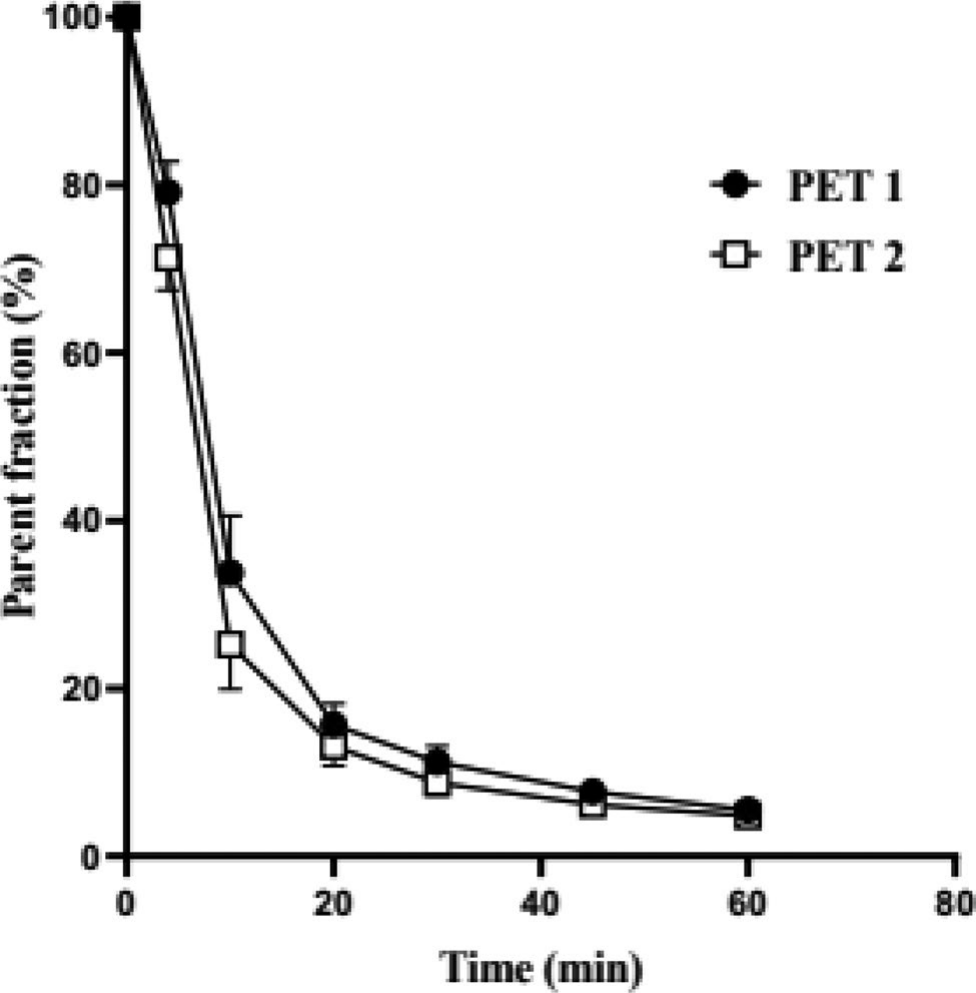
The mean (SD) parent fraction curves for PET1 and PET 2 (*n* = 6)

The metabolite curves were extrapolated to 90 min, using up to 45- or 60-minutes data points, by 3-exp fitting because of later time points having too low signal to noise in high-performance liquid chromatography (HPLC) due to the radioactivity decay and fast metabolism. As demonstrated in Figs. 6 and 10–20% parent compound was present after 20 min injection.

### Test-retest variability and ICC for *V*_T_ in six subjects

Table 3 shows the test-retest variability and ICC in the selected regions for all models. Mean absolute variability was lowest in thalamus and pons and highest in frontal cortex and cerebellum.

The average of absolute test-retest variability of the six subjects for the 12 regions was similar among the models, 6.2 ± 2.7% for 2TC, 6.5 ± 1.3% for Logan, and 6.7 ± 1.1% for MA1. Absolute variability for *V*_T_ calculated by 2TC was lower than those of Logan and MA1 for all the selected regions. The ICC for *V*_T_ was similar among the selected regions and different models. The ICC confidence interval was generally smaller for 2TC compared to that of Logan and MA1.

**Table 3:** Test-retest metrics of absolute variability and ICC for *V*_T_ in selected regions for six subjects

	Absolute variability (Mean ± SD) %				ICC (95% CI)		
Regions	1TC	2TC	Logan	MA1	2TC	Logan	MA1
PONS	5.0 ± 3.6	3.3 ± 2.6	4.6 ±3.1	4.6 ±2.6	0.97 (0.78,1.00)	0.96 (0.54,0.99)	0.96 (0.56,0.99)
THA	5.1 ± 2.5	2.8 ± 1.7	5.5 ± 3.2	5.5 ±3.3	0.98 (0.87,1.00)	0.93 (0.51,0.99)	0.93 (0.51,0.99)
PUT	4.3 ± 2.7	4.2 ±5.5	6.4 ±3.4	6.8 ±2.8	0.93 (0.56,0.99)	0.92 (0.60,0.99)	0.91 (0.57,0.99)
FC	2.6 ± 2.8	6.4 ±5.3	6.9 ±2.7	7.2 ±2.4	0.95 (0.67,0.99)	0.94 (0.21,0.99)	0.94 (0.18,0.99)
CER	3.6 ± 3.7	8.0 ± 5.8	8.2 ±1.7	8.6 ±1.6	0.83 (0.27,0.97)	0.90 (0.28,0.99)	0.90 (0.25,0.99)

**Fig. 7:**
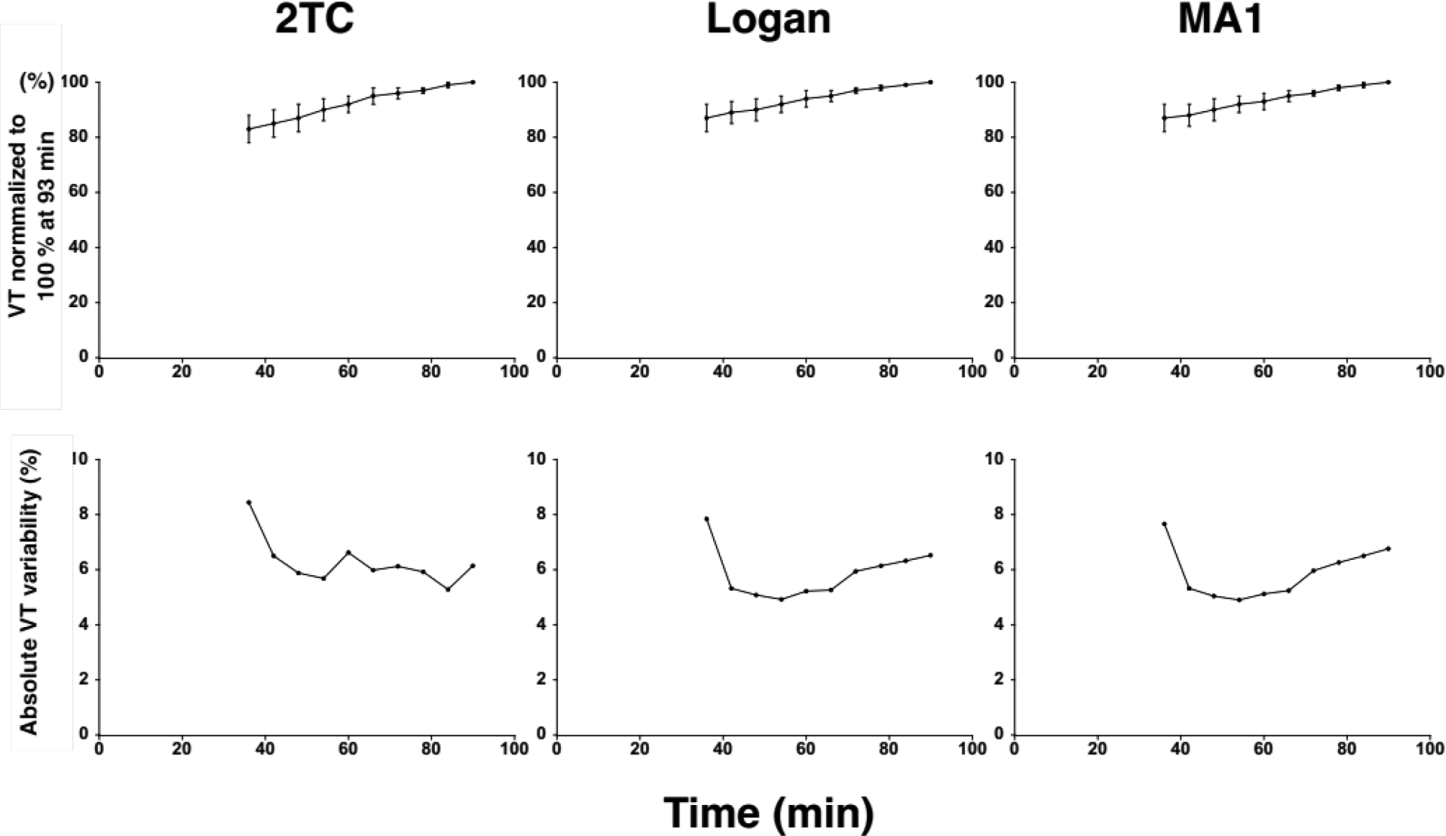
The mean (SD) time stability of *V*_T_ normalized to 100% at 93 min for 2TC, MA1 and Logan models (*n* = 6; PET1; **A-C**); the mean time-stability of absolute *V*_T_ variability (*n* = 6; **D-F**), all plots are for total grey matter

Time time-stability of *V*_T_ is presented in Fig. 7A-C. The *V*_T_ values decreased according to the reduced duration of image analysis. The proportion of *V*_T_ decrease significantly for each duration relative to 93 min. For 2TC, Logan, and MA1 models the *V*_T_ values for 63 min durations were 92%, 93%, and 94% respectively (*p* < 0.0001). Figure 7D shows the time stability of *V*_T_ variability. The absolute *V*_T_ variability did not differ significantly among the different durations of image analysis from 63 to 93 min (*p* > 0.05) (Fig. 7D-F).

## Discussion

In this study, we assessed the quantification and the repeatability of the novel mGlu4 receptor PET radioligand [^11^C]PXT012253 for brain imaging in six healthy volunteers. The administration of [^11^C]PXT012253 in doses of less than one µg was well tolerated. The results demonstrated high brain uptake and *V*_T_ values (range 3.6–6.1 with 2TC across the selected brain regions (Table 2)) as well as good repeatability of the *V*_T_ values (range 2.8-8.0% absolute variability with 2TC among selected regions (Table 2)), providing support that [^11^C]PXT012253 can be used as a mGlu4 PET radioligand in clinical studies.

The brain uptake of [^11^C]PXT012253 reached SUV 4.3–6.3 in the whole brain among the subjects, which is similar to other mGluR radioligands [8, 12, 14, 18]. There was a rapid wash-out, which is favourable for quantification of PET radioligand binding. There was a rapid metabolism of [^11^C]PXT012253, with 10–20% parent compound left 20 min after injection.

The regional time curve of radioactivity among the selected regions showed the highest uptake in thalamus, medium in striatum, and the lowest in cortical regions and cerebellum. Pons showed slightly slow kinetics, most likely due to the substantial white matter tissue component that has slower kinetics. The kinetic property in the WM indicates that in this region there is a slow equilibrating component of [^11^C]PXT012253 (supplements Fig. 1). To the best of our knowledge, there are no mGluR4 receptors in the WM region and the pharmacological profile of PXT012253 does not indicate binding to other targets of relevance. The WM uptake could represent nonspecific binding. In our previous study with [^11^C]PXT012253 in NHPs, the analogue PXT002331 blocked the radioligand’s GM binding by 36% [18], and from the parametric image reported, it seemed that an effect was also observed in the WM. Therefore, off-target binding of [^11^C]PXT012253, for instance to myelin components, cannot be excluded. The specificity of the binding should be further evaluated by blocking studies with suitable analogues of PXT012253.

The data was better described by the 2-TCM than the 1-TCM, based on lower AIC and higher MSC. In the linear graphical analysis, both Logan and MA1 analysis had good fitting to the data and would be the alternative methods considering that graphically derived *V*_T_ values were well correlated to those by 2-TCM analysis despite of a slight overestimation of 6.9% ± 11.2 (Mean ± SD) (Fig. 4). In comparison of graphical models, MA1 showed better identifiability compared to Logan, with lower % COV.

The rank order of the regional TACs and *V*_T_s of [^11^C]PXT012253 in the present clinical study agreed with that previously reported in NHPs [18] and in studies with F-18-labeled mGlu4 PET radioligands [22, 23]. Hence, the regional distribution of [^11^C]PXT012253 binding in the present human brains was considered to represent the regional distribution of mGlu4 receptors.

High repeatability of *V*_T_ values was indicated by the test-retest variability below 10% in all subjects and for all models in the selected regions. Analysis of time stability of *V*_T_ showed significantly, less than 10%, lower *V*_T_ values for 63 min data compared to that of 93 min data in the 2-TCM, Logan and MA1 models (Fig. 7). Test-retest repeatability was insignificantly numerically better at 63 min than at 93 min by Logan and MA1, probably because of the rapid metabolism, the measurement of blood radioactivity data at later timepoints became less reliable due to low signal-to-noise. The low arterial concentration of parent compound at later time-points made the quantification of *V*_T_ likely less reliable. However, there was no statistically significant change of the variability of VT with shorter duration of image analysis. The time stability data suggest that an imaging duration of at 63 min would be preferable for the PET measurement of [^11^C]PXT012253 binding.

Limitations of the study were the low number of individuals examined and the low and unreliable counts in the later arterial blood samples. Increasing the injected radioactivity in future studies might provide better reliability, by increasing the reliability of the measurement of the parent fraction and arterial input function.

## Conclusions

In this study, the mGlu4 PET radioligand [^11^C]PXT012253 was evaluated for the first time in healthy volunteers. We examined the quantification and test-retest reliability of [^11^C]PXT012253 binding to mGlu4 in healthy volunteers. [^11^C]PXT012253 showed a high brain uptake in the grey matter with peak SUV’s ranging between 3.6 and 6.1%. The slow uptake in white matter (supplements) seems not to impact the grey matter data. However, further evaluation with blocking studies is needed. The kinetics of grey matter regions was well described by 2-TCM, and test-retest variability of *V*_T_ was below 10% for selected regions, similar or better than other radioligands without a reference region [24, 25]. The most reliable model for quantifying *V*_T_ estimation based on the ICC values is the 2-TCM. Overall, the present results indicate that [^11^C]PXT012253 is a suitable PET radioligand to measure the mGlu4 receptor in the human brain.

## Appendix 1

Inclusion criteria:

1. Healthy Subjects ag 20–50 inclusive.
2. Body mass index (BMI) of 19 to 30 kg/m2.
3. Normal sMRI scan performed within 3 months, as judged by the investigator.
4. The subject is, in the opinion of the investigator, generally healthy based on assessment of medical history, physical examination, vital signs, ECG, and the results of the hematology, clinical chemistry, urinalysis, serology, and other laboratory tests.
5. Women of childbearing potential and men whose partner is of childbearing. potential must be willing to ensure that they or their partner use effective contraception (see Sect. 5.6.8) during the trial and for 3 months thereafter.
6. A propensity to tolerate confined spaces for prolonged periods.
7. Suitability for radial and/or brachial artery blood sampling and cannulation.

Exclusion criteria: Involvement in the planning and/or conduct of the study.

8. Previous enrolment (subject dosed) in the present study.
9. History of clinically significant cardio-or cerebrovascular, pulmonary, renal, hepatic, neurological, mental or gastrointestinal disorder or any other major disorder that may interfere with the objectives of the study, as judged by the investigator.
10. The subject has 1 or more clinical laboratory test values outside the reference range, which in the opinion of the investigator are of clinical significance.
11. Any finding of significance on sMRI scans as judged by the investigator.
12. Screening supine blood pressure > 140 mm Hg (systolic) or > 90 mm Hg (diastolic), following at least 5 min of supine rest. If blood pressure (BP) is > 140 mm Hg (systolic) or > 90 mm Hg (diastolic), the BP should be repeated two more times and the average of the three BP values should be used to determine the subject’s eligibility.
13. The subject has a resting pulse < 50 or > 100 bpm at the Screening Visit.
14. The subject has a QTc interval > 430 ms (Bazett’s or Fridericia’s correction) at the.
15. Screening Visit or at the Baseline Visit, as calculated by the ECG equipment and evaluated by the investigator. The ECG may be repeated if any of the values are out-of-range or abnormal.
16. Inability to perform sMRI as judged by the investigator, e.g., due to a metal object in the body or claustrophobia.
17. History of psychiatric, neurological and any acute or chronic disease/condition or surgery that may interfere with the objectives of the study, as judged by the investigator.
18. Administration of any investigational product within 3 months before the start of the present study.
19. Habitual use of nicotine products and addictive substances (see 5.6.3).
20. Known or suspected drug, alcohol or other abuse, or positive Serum drug screen which may interfere with the study objective.
21. Use of CNS active drugs and/or NSAIDs (Non-Steroidal Anti-Inflammatory Drugs) 1 month prior to the first PET examination.
22. The subject trains/exercises intensively, for example, for a marathon or triathlon, or at a competitive level.
23. The subject has worked shifts, including night duty, or has travelled across > 3 time zones < 2 weeks prior to the first dose administration.
24. History of severe allergy/hypersensitivity or ongoing allergy/hypersensitivity as judged by the investigator.
25. Any previous PET measurements for scientific purposes.
26. The subject is exposed to significant levels of ionizing radiation at work.
27. The subject has undergone any clinical procedures involving significant exposure to radiation (excluding dental X-ray and common X-rays of the chest or extremities).
28. The subject has received radiolabelled material < 12 months prior to the Screening Visit.
29. The subject suffers from claustrophobia or needle phobia.
30. The subject has implanted or embedded metal objects, prostheses, or fragments in the head or body that would present a risk during the sMRI scanning procedure or have worked with ferrous metals either as a vocation or hobby (for example, as a sheet metal worker, welder, or machinist) in such a way that might have led to unknown, indwelling metal fragments that could cause injury if they moved in response to placement in the magnetic field.
31. The subject is, in the opinion of the investigator, unlikely to comply with the protocol or is unsuitable for any reason.
32. The subject is pregnant or breastfeeding.

## Electronic Supplementary Material

Below is the link to the electronic supplementary material

Supplementary Material 1

## Data Availability

The datasets generated during and/or analysed during the current study are available from the corresponding author on reasonable request.
