# Supplementary material for: Test-retest properties of [11C]PXT012253 as a positron emission tomography (PET) radiotracer in healthy human brain: PET imaging of mGlu4
Source: EJNMMI Res. 2025 Jun 14;15:71. doi: 10.1186/s13550-025-01266-y (PMC12167413; doi:10.1186/s13550-025-01266-y)
Supplement: Supplementary file 1 — Supplementary Material 1 [file 13550_2025_1266_MOESM1_ESM.docx]

**Figure 1 Time activity curves of regional radioactivity in the brain after i.v. injection of [^11^C]PXT012253 in a healthy volunteer (No 1). GM: grey matter; WM: white matter.**
